# Research on antipsychotics in India

**DOI:** 10.4103/0019-5545.69261

**Published:** 2010-01

**Authors:** Ajit Avasthi, Munish Aggarwal, Sandeep Grover, Mohd Khalid Rasheed Khan

**Affiliations:** Department of Psychiatry, Postgraduate Institute of Medical Education and Research, Chandigarh, India; 1 Department of Pharmacology, Shadan Institute of Medical Sciences, Hyderabad, India

**Keywords:** Antipsychotic, research, India

## Abstract

Antipsychotic as a class of medications became available for treatment of various psychiatric disorders in the early 1950’s. Over the last 60 years many antipsychotics have become available. In line with the west, Indian researchers have evaluated the efficacy of antipsychotics in various conditions. Additionally, researchers have also evaluated the important safety and tolerability issues. Here, we review data originating from India in the form of drug trials, effectiveness, usefulness, safety and tolerability of antipsychotics. Additionally, data with respect to other important treatment related issues is discussed.

## INTRODUCTION

When one looks at the history of psychiatric treatments, prior to availability of electroconvulsive therapy (ECT), measures like magic, restraints, blood letting, emetics, purgatives, surgical operations on various organs, removal of foci of infections, vaccines and endocrines were tried as treatment options for schizophrenia. Therapies like insulin coma and electroconvulsive therapy became available.[1] However, the era of pharmacotherapy for treatment of schizophrenia started with use of chlorpromazine by Delay and Deniker for the treatment of patients suffering from schizophrenia in early 1950’s. Over the next half century, a large number of drugs have been evaluated and marketed as antipsychotics. This class of drugs also helped in understanding the neurobiology of schizophrenia to some extent. This class of drug has also changed the attitude of the clinicians towards the expected outcome of the disorder.

India, as a country was not isolated from all these developments. Over the years many classes of antipsychotics have become available in India, some of which have stood the test of time and are still in use and some are no more marketed or are no more favorite of the clinicians. Research focusing on the usefulness of psychotics in India has more or less followed the trends in the West; however, some of the newer antipsychotic drugs which are currently marketed have not been evaluated as thoroughly as others. The pharmaceutical industry and the policy of the government have ensured that these medications are available at a reasonable price.

This review focuses on research done on various anti psychotics in India. For this a thorough internet search was done using key words like India, antipsychotics, name of each antipsychotic, efficacy, effectiveness, usefulness, tolerability, side effects, metabolic syndrome, weight gain, prescription, cost in various combinations. Various search engines like PUBMED, GOOGLE SCHOLAR, SCIENCEDIRECT, SEARCH MEDICA, SCOPUS, and MEDKNOW were used. In addition, a through search of all the issues of Indian Journal of Psychiatry available online was done. Hand search of some of the missing issues was also attempted and this yielded a few more articles. We have excluded review articles which we felt did not reflect the Indian scenario to a large extent or did not cover the available Indian data. Data from the animal studies that originated in the form of case reports and studies published only as abstract have not been included.

## EFFICACY OF ANTIPSYCHOTICS IN SCHIZOPHRENIA

### Efficacy of first generation antipsychotics in schizophrenia [Table 1]

Twenty eight studies which have evaluated the efficacy of first generation oral antipsychotics in schizophrenia have been published.[2–29] Of these, most have been open trials, some have been double blind randomized controlled trials and only a few have compared active drug with placebo. Some have also followed the cross over design with intermediate drug free period. Some of the initial trials included subjects who had not responded to previous pharmacological or somatic treatment and were suffering from chronic schizophrenia. Further, some studies have included treatment refractory subjects and others have recruited only those subjects who responded to some medication in the past. The sample size has varied from 15 to 145 subjects and the duration of trials have varied from eight days to six months, but most have evaluated the subjects in 4-12 weeks. Most of the trials included subjects with acute symptoms, however, one trial evaluated the efficacy of penfluridol and haloperidol in the maintenance phase and another included subjects who, on baseline, had shown at least 50% response on BPRS to the previous antipsychotic medication.[29]

**Table 1:** Efficacy of first generation antipsychotics in schizophrenia

Authors	Duration (weeks)	Sample size/scale/design	Medication (s)	Dose (s) (mg)	Outcome	Side effects
Doongaji *et al*.[2]	6	N = 18; Open Trial	Prothi pendyl	Phase I- 120-240 Phase II- 160	2 out of 8 subjects in the psychosis group (who were administered drug im) showed significant improvement, 3 cases showed some improvement and 3 cases showed no improvement	The optimum dose of the drug ranged between 120 mg/day and 160 mg/day Doses above 160 mg/day caused more sideeffects, and dose reduction was needed in 4 cases Side-effects were increased if the drug was prescribed as single doses exceeding 40 mg 5/8 subjects who were administered dose im had significant fall in blood pressure
Sarada Menon[3]	8	N = 20; Catatonic Schiz DBPCT	PCPZ Vs. PBO	100	2 out of 10 subjects who received PCPZ were remarkably improved, 1 showed favorable improvement and 5 showed appreciable change in behavior. No change was observed in 2 subjects 1 case of periodic psychosis improved on PBO The earliest change in behavior in the PCPZ group was decrease of withdrawn behavior and it was observed by 4-5 weeks	Drug was discontinued in one subject because she became cold and clammy and she refused to take food Another patient developed hypotension, cold extremities, EPS and developed excitement There was no change in blood counts and there was no evidence of hepatic damage
Damania and Masani[4]	8 days-4months	N = 20; Open label Schiz.	TRZ	30-300	18 out of 20 subjects with Schiz improved, of which 1 was considered as cured	Drowsiness and giddiness No serious S/E were noted
Kothari[5]	4	N = 15; (12 Schiz) Open label, Treatment Refractory	FFP oral	2.5-20	1 patient showed marked improvement, 2 subjects with catatonic stupor showed mild improve ment, in 4 patient hyperactivity worsened and they became violent, 8 subjects showed no change	4 subjects became extremely disturbed and violent 5 subjects showed EPS (excessive salivation, rigidity, and tremor but these were mild)
Bhaskaran[6]	6	N = 35; Open label, Schiz.	TPZ	90	Of 28 Schiz subjects- 3 subjects recovered, 11 subjects improved markedly, 8 improved and 6 remain unchanged	The Akinetic-hypertonic syndrome was noticed in 25 cases Hyperkineto-Hypertonic Syndrome was noticed in 16 subjects Both these syndromes improved with antiparkinsonian medications, ankinetic type improved more than hyperkineto- hypertonic type No serious CVs. adverse effects No evidence of significant neutropenia or hepatic damage
Thomas and Narayanan[7]	12	N = 10; RCT, Koh’s block design, pass along test, MMPI, TAT	TFP Vs. UCPZ	TFP-5-15 UCPZ-200	Of the 6 subjects receiving TFP, 1 showed marked improvement, 3 showed moderate improvement, 1 showed slight improvement and 1 died after 7 weeks of trial, (cause of death not known, had features s/o vitamin B deficiency) Of the UCPZ group, 2 showed moderate level of improvement, 1 showed slight improvement and 1 showed no improvement A trend of improvement in intelligence, however, the difference was not significant between the 2 groups	1 out of 6 TFP pt had S/E- masked faces, rigidity, drooling of saliva, improvement in S/E with decrease in dose and adding THF UCPZ group- giddiness Periodic blood and urinary check-ups in both groups showed no significant changes
Jetley[8]	6	N = 16; (Schiz-10; Mania-6), Open label	HPL	Mania–1.5-4.5 Schiz- 6-9	All subjects with schizophrenia showed improvement in sphere of over activity and verbal aggression towards others in environment and also in sphere of social contacts. In 4 subjects the paranoid delusions melted away and there was improvement in inter-personal relationship	Subjects developed EPS which improved with THF 2 subjects developed intractable motor restlessness which responded to phenergan in one
Dube and Mathur[9]	4	N = 35; Open label	TRZ	300	10 subjects showed good improvement, 5 showed moderate improvement, 8 slight improvement and 12 did not show any improvement Catatonic excitement responded best and most swiftly Symptoms of a better response included-irritability, excitability and abusiveness, overactivity spontaneous talks, mutterings and incoherency Symptoms corresponding to the fundamental symptoms of schizophrenia (thought disorder, thought block, emotional blunting, odd mannerisms, carelessness, poor judgment and tendency to roam aimlessly) responded least Better results were seen in early illnesses and lower age group Females showed less improvement than males, but the difference was not significant Patient with mental sub normality showed no improvement	S/E- Weight gain, tachycardia, drowsiness, lability of pulse, hypotension, stuffiness of nose, giddiness, dryness of mouth, stuffiness of tongue, excessive salivation, loss of appetite, vomiting, metallic taste, weakness, subnormal temperature, feeling of intoxication.
Narayan *et al*.[10]	20	N = 20; Comparative trial	PCPZ Vs. CPZ	PCPZ-100 CPZ-200	In the PCPZ group 5 subjects were rated as much improved, 3 as moderately improved, 1 as slightly improved and 1 remained as before In the CPZ group, 3 subjects were rated as much improved, 4 as moderately improved, 1 slightly improved and 2 had no change in their status In majority of the cases, definite changes were observed between the 5^th^ and 6^th^ week after starting of the drugs, the earliest sign was the shedding of withdrawal, and they were more communicable and manageable PCPZ seemed to increase drive and affect in chronic schizophrenics, whereas CPZ was found to be about 20% less effective	3 of the 10 PCPZ subjects developed EPS with in first week and required treatment with phenergan No hematological S/E or liver dysfunction
Teja[11]	14 months	N = 25 Schiz Open label PSSR	Thiopro perazine	5-25	6 subjects recovered, 5 markedly improved, 3 improved and 11 maintained same. 1 patient who had inadequate trial showed marked improvement Best results were seen in paranoid and catatonic groups There was no evidence of any relation between therapeutic response and occurrence of S/E	No abnormality was detected on hematological, urinalysis or liver function test S/E noted were weakness, tremors, rigidity, agitation, anxiety, visual accommodation difficulty, insomnia, headache, depression, masseter spasm, tachycardia, sialorrhoea, petrified dejected look, opisthotonus, parkinsonian facies, excessive sweating, transient urinary retention, elation, speech difficulty, facial spasm, anorexia, indifference, burning sensation, dizziness
Sarada Menon and Haneef Badsha[12]	1 year	N = 30 Chronic Schiz, Rating on Behavior chart, DBRCT cross over design	Thiothixene vs. TFP vs. PBO	Thioth ixene - 15-30 TFP-15-30	In controlling delusion and hallucinations, thiothixene was better than TFP In controlling over talkativeness and occupational activity TFP was better than thiothixene On analysis of all symptoms, it was found that 44.2% of improvement was due to thiothixene, 35.3% was due to TFP and 20.5% was due to PBO 27 subjects showed improvement with thiothixene, 24 subjects showed improvement with TFP and 8 with PBO out of 30 subjects	EPS, autonomic symptoms, and GI S/E were noted No S/E was serious enough to warrant discontinue No abnormality was noted on hematological investigations, LFT, RFT and urine analysis
Kishore *et al*.^13^]	3 months; After 3 months, each patient was put on CPZ 300 mg/day and followed for 3 months	N = 60 Chronic Schiz, PSSR DBCT	Thiothixene vs. triflupro mazine vs. CPZ vs. TFP vs. PCPZ vs. Thiopro perazine	Thiot hixene-30 Triflupro mazine-150 CPZ-600 PCPZ-150 TFP-30 Thiopro perazine-30	Significant improvement was seen in 8 subjects on TFP, 7 on PCPZ, 5 each on thiothixene and thioproperazine and 4 each in CPZ and triflupromazine group Drugs have different degrees of improvement in various symptoms. In behavioral disturbances 80% subjects in PCPZ group showed improvement, 60 % each in TFP, thiothixene and thioproperazine, and 50% in both CPZ and triflupromazine group In thought disorder, 70% in PCPZ group, 40% in TFP, CPZ and thiothixene group, 30% in thioproperazine group and 20% in triflupromazine group In the affective symptoms, improvement was seen in 70% in PCPZ group and TFP group, 50% each in thiothixene, thioproperazine and triflupromazine group and 30% in the CPZ group In the symptoms of sleep, appetite and sexual disturbances, 100% improvement in PCPZ, TFP, CPZ and triflupromazine group and 75% in both thiothixene and triflupromazine	EPS (rigidity, tremors, akathisia, dystonia) CNS (giddiness, insomnia, feeling of weakness in limbs Autonomic (hypersalivation, constipation, urinary frequency) Endocrinological (gynecomastia) Toxic (jaundice, skin rash/Pruritus) All these drugs caused a fall in BP in 50-90% of the subjects
Bagadia *et al*.[14]	4	N = 145 Open label, Schiz	FFX	1.5-9	29 subjects dropped out before completion of 2 weeks of trial Out of 116 subjects who continued treatment for 2-4 weeks, 54 showed significant improvement (46.5%). Significant improvement was observed in 46.1% of the subjects in drive deficit group and in 26.8% of the subjects in non-drive deficit group. Significant improvement was highest in the catatonic group (12/18 i.e. 66.6%) 8/19 (42.1%) subjects of chronic type had significant improvement.	Drowsiness (commonest S/E), tremor, EPS, giddiness, generalized weakness, restlessness, dryness of month, constipation, nausea, increased appetite, excessive perspiration
Bagadia *et al*.[15]	4	N = 100 Open label	TFD	1-4	24 subjects dropped out before completion of 3 weeks of trial	EPS (Tremors, spasms and rigidity, oculogyric crisis, slurred speech), palpitations, chest-pain, dryness of mouth, constipation, drowsiness, fatigue, weakness, skin rash.
					50 out of 76 subjects (65.8%) (Who completed three or more weeks of treatment) showed significant improvement.	
					Subjects with paranoid schizophrenia responded best	
					None of the cases of schizoaffective and pseudoneurotic schizophrenia improved. In other types (simple, hebephrenic, chronic undifferentiated, acute undifferentiated and mixed type), the rate of improvement was on an average 61%.	
					Subjects with illness duration less than 1 year showed maximum improvement.	
Ramachandran and Sarada Menon[16]	6	N = 50 Consecutive sampling; Schiz DBRCT, QPSS, WBRS	TFD Vs. PBO	15	88% of the patients on TFD showed marked improvement and 8% showed moderate improvement compared to 8% and 23% respectively on PBO and the difference was statistically significant.	EPS
					Patients on TFD showed significantly better improvement in thought disorder, emotional disturbances, catatonic symptoms, ideas of reference, persecutory and grandiose delusion and auditory, visual, olfactory, gustatory and tactile hallucination.	No changes in laboratory investigations
					Patients on TFD showed significantly better improvement in thought disorder, emotional disturbances, catatonic symptoms, ideas of reference, persecutory and grandiose delusion and auditory, visual, olfactory, gustatory and tactile hallucination.	
					Improvement in reality orientation, judgment, and insight were also seen.	
Vyas and Bapana[17]	6	N = 40	Go 3315	300-600	65% of the subjects improved while 35% did not show improvement.	S/E were not reported by the authors
					Symptoms of insomnia, automatic obedience, anxiety, passivity feelings, stereotype behavior, mannerism, suspiciousness completely recovered.	
					Symptoms of excitement, negativism, impulsive behavior, inappropriate behavior, paranoid delusion, apathy and other delusions showed marked improvement.	
					Lack of volition and incoherence were minimally improved and there was no improvement in depersonalization.	
Kishore *et al*.[18]	3 months	N = 60 Chronic Schizophrenia, PSSR, DBCT	TFD Vs PCPZ Vs Thioxanthene	TFD-6 PCPZ-150 Thioxanthene-30	All three drugs were effective for psychotic symptoms and symptoms in the domains of sensorium, behavior, thought and emotional disturbance.	S/E were maximum in case of TFD followed by thioxanthine and were least in the PCPZ group
					There was no significant difference between the three drugs in overall improvement in psychopathology and between PCPZ and thioxanthine in the subscales of sensorium, behavior, thought and emotions.	
					However, in the subscale of sensorium, PCPZ was better than TFD, and both PCPZ and thioxanthine were better than TFD in the subscale of emotional disturbances.	
					The dosages used in the present study are effective dosages in majority of the subjects.	
Kishore and Dhillon[19]	3 months	N = 40 females Schiz, Open label, MSQ	PMZ	1-5	11 subjects showed slight improvement, 16 showed moderate improvement, 11 showedd good improvement, 1 patient had excellent improvement, and 1 patient was unchanged or slightly worse.	There was no abnormality on haematological parameters or urinalysis
					The psychotic symptoms were reduced by 13.5%.	Mild EPS in form of tremors, rigidity, drooling nausea and vomiting, akathisia was seen.
					The ward bearing and behavior improved by 21.0%	Severe EPS occurred in 1 patient who had tremor, rigidity, akathisia and excessive salivation.
					Symptoms which improved considerably included emotional withdrawal, hostility, tension, anxiety and blunted affect.	1 subject developed somatic concern and 3 subjects had suspiciousness while on treatment.
					There was also appreciable improvement in disinterest in appearance, reluctance to work, social withdrawal, lack of leisure interests, and misbehavior at meals, this helped them to get engaged in work.	
Sarada Menon *et al*.[20]	8	N = 40 Chronic Schiz, BPRS, QPSS Controlled cross over trial (8 weeks of drugs followed by 6 weeks of washout and then 8 weeks of other drug)	PMZ Vs. FFP	PMZ-8 FFP-10	It was observed that symptoms became worse while the subjects were on other medication and improved significantly while on PMZ or FFP.	On PMZ 1 patient developed EPS and 3 developed insomnia, the same was 4 patients and 1 patient respectively for FFP.
					On QPSS, improvement was noticed for patients on PMZ and FFP.	
					Both PMZ and FFP were found to have a highly significant therapeutic effect.	
Bagadia *et al*.[21]	12	N = 61; Open label, Treatment responsive Schiz.	PMZ	1-4	After the subjects were shifted to PMZ, 68% subjects maintained improvement, 26% had further improvement while 12% of the subjects worsened.	Oculogyric crisis, salivation, rigidity, tremors, restlessness No significant change was seen in hemogram, liver function tests, blood urea, blood sugar and urine routine examination
De Sousa and Nayani[22]	6	N = 50; Schiz Controlled trial	TFD vs. TFP	TFD-3 TFP-15	17 subjects showed significant improvement in the TFP group, while 15 in TFD group showed significant improvement TFD group more improvement in excitement and insomnia TFP had better control of delusions and hallucinations Both the drugs were equally efficacious in controlling concentration and irrelevant speech	Mild S/Es seen in both the groups: Drowsiness, constipation, blurring of vision, dryness of mouth, EPS (rigidity and tremors), difficulty in micturation, fatigue, rash TFD: Serious EPS (N = 2), skin rash (N = 1)
Mahal and Jana kiramaiah[23]	6 months	N = 62 Chronic Schiz, DBPCT cross over design, SMSQ	PMZ vs. PBO	2-4	In the first three month trial period there was greater improvement in the PMZ group as compared to the PBO group In the second three-month period there was no improvement in both the groups, rather psychotic symptoms and ward behavior worsened	EPS and sedation seen initially with PMZ
Channa basavanna *et al*.[24]	3	N = 36 Open label Chronic Schiz. WPRS, LFFMBS	TFD	1.5	25 (69%) subjects showed improvement (19 showed marked improvement, and 7 showed fair improvement) The improvement was significantly more for subjects without family history of schizophrenia On WPRS, mean scores decreased on depressive state, schizophrenic excitement and hebephrenic syndromes On LFFMBS, there was significant improvement in behavioral response to meal, attention to dress and personal cleanliness, speech and toilet behavior and response to psychiatrists, psychologists, nurses, etc	14 subjects developed EPS, which responded to either dose reduction or adding antiprakinsonian medications No significant side effects such as, drowsiness, orthostatic hypotension, GI disturbances, skin reaction etc, were noticed
Sharma and Dutta[25]	4	N = 34; Schiz, DBPCT (5 stage trt- withdrawal of previous trt to rando mization)	PMZ vs. PBO	1-5	Of the 19 patients treated with PMZ, 11(57.89%) showed moderate to marked improvement, no subject improved in PBO group and the difference between the 2 groups was significant.	Four patients had drowsiness and weakness, 1 patient became more aggressive with increased psychomotor activity, one patient developed severe tremors, rigidity and oculogyric crisis
Bagadia *et al*.[26]	2 phases of 3 month each	N = 50;DBCT cross over trial Schiz with optimal level of improve ment (>50% on BPRS were recruited), BPRS	PMZ vs. TFP	PMZ -2 TFP-10	Equal number of subjects dropped out of either drug There was no statistical difference in the number of subjects who showed further improvement, maintained same or worsened further No significant difference was noted between the two groups on improvement on BPRS at 3 months or improvement between 3 and 6 months	2 subjects with TFP had EPS (parkinsonian features and akathesia) 1 patient on PMZ had tremor and hypotension
Dube and Sethi[27]	6	N = 60 Schizophrenia DBCT cross over trial MBPRS, CGIS	Li vs CPZ		On modified BPRS, overall CPZ was better than Li for majority of the items for most of the time On CGIS both Li and CPZ showed significant improvement from the 2^nd^ week onwards From week 1-4, CPZ was better than Li in improvement in severity of illness After 1^st^ and 3^rd^ week of treatment, CPZ was better than Li in global improvement	Not reported by authors
Sethi and Bhiman[28]	4	N = 30 Feighner’s criteria, Schiz, BPRS, Side effects symptoms checklist, CGIS, DBCT	TFP vs TFP-THF	TFP-5 TFP-THF (5/2)	The combination of THF does not reduce clinical efficacy of TFP.	The incidence of side effects is lower in the group receiving the combination (except dryness of mouth) whereas rigidity and muscle spasm are entirely absent.
Channabasavanna and Michael[29]	12 weeks (mainte nance phase)	N = 30; DSM-III Chronic Schiz DBCT, SAPS, SANS, SAS	Penflu ridol vs. PBO vs. HPL	60 mg/week	Penfluridol was significantly better than placebo for maintenance therapy In case of worsening of condition, the patient was given HPL Only 1 patient in the Penfluridol group required supplemental haloperidol while 12 among the placebo group did Penfluridol was as efficacious as HPL for maintenance therapy	EPS were noted No serious S/E were encountered General physical condition and blood chemistry, hematological investigations and urine analysis not revealed any abnormality

Schiz - Schizophrenia; DBPCT - Double blind placebo controlled trial; PCPZ - Prochloperazine; PBO - Placebo; EPS - Extrapyramidal symptoms; TRZ - Thioridazine; S/E - side effect; FFP - Fluphenazine; TPZ - Thioproperazine; CVs. - Cardiovascular system; RCT - Randomized controlled trial; MMPI - Minnesota multiphasic personality inventory; TAT - Thematic apperception test; TFP - Trifluoperazine; UCPZ - Unichlorpromazine; THF - Trihexyphenidyl; HPL- Haloperidol; CPZ- Chlorpromazine; PSSR - Psychotic symptom severity rating chart; DBRCT - Double blind randomized controlled trial; GI - Gastrointestinal; LFT-Liver function test; RFT - Renal function test; DBCT - Double blind controlled trial; FFX - Fluphenthixol; TFD - Trifluperidol; QPSS - Quantification of psychotic symptom severity; WBRS - Wing’s behavior rating scale; CNS - Central nervous system; PMZ - Pimozide; MSQ - Mental status questionnaire; SMSQ - Special mental status questionnaire; WPRS - Wittenborn psychiatric rating scale; LFFMBS - L-M fergus falls behavior schedule; BPRS - Brief psychiatric rating scale; Li- lithium; MBPRS - Modified brief psychiatric rating scale; CGI-S - Clinical global impression severity; SAPS - Scale for assessment of positive symptoms; SANS - Scale for assessment of negative symptoms; SAS - Simpson and angus rating scale for extrapyramidal side effects

Except for some of the trials in the 1960s and early 1970s, the efficacy and side effects were measured on some rating scales. All these trials have not used the dropouts in their final analysis of data. Data from all these trials can be summarized as: Typical antipsychotics are useful for treatment of schizophrenia, typical antipsychotics are more efficacious than placebo in the treatment of schizophrenia, improvement is seen more in paranoid and catatonic symptoms, aggression and less so in depressive symptoms; addition of trihexyphenidyl doesn’t reduce the efficacy of antipsychotic like trifluperazine and extrapyramidal symptoms are common with typical antipsychotics.

### Efficacy of injectable depot first generation antipsychotics in schizophrenia [Table 2]

Nine trials have evaluated the efficacy of depot antipsychotics in schizophrenia.[30–38] Some of these trials have just focused on short term outcome (2-4 weeks), whereas others have evaluated the outcome after six months. One trial followed up the subjects for 1-3 years.[37] In most of these trials subjects included were those who were non-complaint to oral medications and had frequent relapse. All these were open label and did not use a comparator group, except for the trial by Bagadia *et al*.[36] that compared subjects on fluphenazine decanoate with subjects who has been receiving either chlorpromazine or trifluperazine. All these studies have shown that depot antipsychotics are useful in the management of schizophrenia in acute phases and also for maintenance treatment.

**Table 2:** Efficacy studies of injectable depot antipsychotics in schizophrenia/psychosis

Authors	Duration (in weeks)	Sample size/scale/design	Medication(s) and dose (s) in mg	Outcome
Iyer *et al*.[30]	2	N = 30; Schiz, Open label Jenkin’s rating scale	FFZ enanthate 12.5-62.5	Improvement (>50% reduction on Jenkin’s rating scale) Out of 28 subjects, 18 improved Drug had maximum effect on symptoms of withdrawal, guarding, cooperation, mannerisms, hallucinations, motivation and post-hospitalization goals Physical, anxiety and depressive symptoms had not responded
Bagadia *et al*.[31]	4	N = 50; Schiz, Open label	FFP Decanoate 25-150 mg/month	78% of the subjects improved (6% completely improved, 38% markedly improved, 34% moderately improved) All subtypes of schizophrenia including the chronic undifferentiated type showed good response Subjects with poor prognosis had 80% improvement Shorter duration of illness seems to have been benefited the most Paranoid delusion, auditory hallucination, excitement and apathy responded best to the drug Follow-up was done for 8 weeks after one month of drug trial was over
Bagadia *et al*.[32]	4 weeks to 6 months	N = 40; (Chronic Schiz) and N = 78; (schiz)	FFZ Decanoate Total dose 50-500mg	Of 26 subjects of chronic schizophrenia, who completed trial for 4 weeks, 20 showed significant improvement 66 (84.6%) of the subjects who took treatment for 1 to 6 months classified as good prognosis schizophrenia improved significantly
Jha and Bhaskaran[33]	Duration not mentioned (7 to 54 injections)	N = 112; Schizophrenia, Open trial	FFZ 25 mg/week	3.6% of the subjects recovered, 3.6% had marked improvement, 25% were rated as improved, 22.3% were rated as manageable, there was no change in 41.8% of subjects, while 1.8% each worsened and discontinued treatment and 0.9% of subjects died Of the 28 subjects who improved, 4 subjects continued to develop psychotic symptoms periodically despite being under medication while 9 subjects showed relapse Paranoid type of schizophrenia responded best while hebephrenic type responded least Symptoms which worsened were depression, guilt feelings and agitation FFZ was found to be costlier than CPZ, when costs of antiparkinsonian medication was combined with that of FFZ
Gehlot *et al*.[34]	20	N = 30; Schiz (Subjects who had not responded to oral phenothiazines, butyrophenones, ECT, relapsed or had non-compliance)	FFP decanoate25 mg/2-3 weekly	60%subjects responded favorably Symptoms which improved-formal thought disorder, ideas of reference and control, delusion of persecution, grandeur and infidelity, disturbance of affect e.g. inappropriate affect, apathy, irritability), auditory hallucinations, uncooperativeness, aggressiveness, and impulsive behavior, poor self care Symptoms which had not responded included- mutism, mannerism, lack of initiative, muttering, hypochondriasis, severe preoccupation, silly giggling
Bagadia *et al*.[35]	3 months	N = 27; Schiz, Open trial, BPRS	Pipothiazine palmitate 50 mg/weekly	76% of the subjects showed significant improvement at end of 1 month; further significant improvement occurred between 1 and 3 months.
Bagadia[36]	4 months	N = 83; ICD-8 Schiz, BPRS, social adjustment scale, work efficiency evaluation scale	FFP Decanoate started at 6.25/week increased weekly vs. CPZ/TFP	54% of the subjects made >50% of the expected visits 83% of the subjects improved on BPRS, 85% on social adjustment scale and 94% on work efficiency evaluation scale
Shukla[37]	1-3 year	N = 65; Chronic Schiz patients with frequent relapses despite treatment with neuroleptics and/or ECT	FFP Decanoate 25 mg/3 week	FFP was found to be effective in 25 (62.5%) of the subjects who completed the trial Response was better in patients with onset of illness after 20 years of age, illness duration 2-5 years, with a diagnosis of paranoid and undifferentiated subtype, followed by catatonic subtype Patients with hebephrenic and simple subtype showed poorer response
Varma and Kulhara[38]	24 (Maintenance phase)	N = 33; DSM-III Schiz, Open trial, BPRS, SANS, CGI, AIMS, DOTES, ESRS	HPL Decanoate 50-300/4 week	At the end of the study period, 20 subjects had shown much improvement and 10 had minimally improved Significant reduction in severity of illness score was noted from the 8^th^ week onwards Significant improvement on CGI was observed from the 4^th^ week onwards Significant reduction in BPRS scores was noted from the 2^nd^ week onwards There was significant reduction in the SANS score

Schiz - Schizophrenia; FFZ - Fluphenazine enanthate; FFP - Fluphenazine decanoate; ECT - Electroconvulsive therapy; BPRS - Brief psychiatric rating scale; CPZ - Chlorpromazine; TFP - Trifluoperazine; SANS - Scale for assessment of negative symptoms; CGI - Clinical global impression scale; AIMS - Abnormal involuntary movement scale; DOTES - Dosage record and treatment emergent symptom scale; ESRS - Extrapyramidal symptoms rating scale

### Efficacy studies of first generation antipsychotics vs. electroconvulsive therapy/electroconvulsive therapy and first generation antipsychotics in schizophrenia [Table 3]

Thirteen studies have compared typical antipsychotics with either electroconvulsive therapy (ECT) alone or a combination of ECT and typical antipsychotic medication.[1,39–50] All studies have specified the diagnosis as schizophrenia, except for one study, in which the diagnosis was mentioned as functional psychosis.[45] A common theme which emerges from these studies is that typical antipsychotic, alone or when used in combination with ECT, produces similar response rate in short term, but addition of ECT leads to a faster response. The only study which evaluated the efficacy of ECT and chlorpromazine in treatment resistant schizophrenia (TRS) showed that augmentation with ECT in TRS may be a worthwhile option.[50]

**Table 3:** Comparative efficacy studies of first generation antipsychotics vs. electroconvulsive therapy/electroconvulsive therapy and first generation antipsychotic in schizophrenia

Authors	Duration (in weeks)	Sample size/scale/design	Medication (s) in mgs	Outcome
Dutta Ray[39]			CPZ vs. CPZ + ECT vs. ECT	Combination of these two forms of treatment gave better results than when either of their use singly
Chatterjee and Bhushan[40]	1-4 month	N = 76; Open label	TRZ + ECT + IMN (in new cases) vs. TRZ	20 out of 25 new cases showed considerable improvement in 2-3 weeks 27 out of 51 old cases showed good response
Dutta Ray and Kapur[41]	Follow-up 6-18 months	N = 200; Psychotic subjects	CPZ + ECT	Better chances of response to combination treatment, if the duration of illness is less than 1 year, patients are young and have an acute onset of illness 37% of the subjects were classified as cured, 41.5% as relieved and 21.5% as unrelieved Family history of mental illness was poor prognostic factor whereas gender and past h/o psychiatric illness had no bearing on the treatment response
Bagadia *et al*.[1]	4	N = 300; Schiz Group matching on the basis of prognosis	IST vs. ECT 6-10 vs. CPZ 600-2400 vs. TFP 15-60 TFD 2-6 vs. FFX 3-15	Improvement in the ECT group was better and faster than all other groups 90% of the subjects improved with ECT (80% improved completely, 2% improved markedly, 8% improved moderately) compared to 60-78% in other groups In the ECT group all the subjects with very good, good and fair prognosis improved completely
Janakiramaiah and Subbakrishna[42]	6	N = 44; SBRCT, RP, CGIS	CPZ 300 CPZ 300 + ECT	There was no significant difference between the two group on RP scale or CGIS at the end of 6 weeks ECT + CPZ group was significantly better than CPZ alone on the RP scale positive and negative scores at the end of 2^nd^ week
Janakiramaiah *et al*.[43]	6	N = 60; RDC Schiz, SBRCT, Consecutive sampling, BPRS, ESRS, CGI	CPZ 300 mg vs. CPZ 500 mg vs. CPZ 300 mg + ECT vs. CPZ 500 mg + ECT	CPZ 300 mg better than CPZ 500 mg/day ECT/CPZ combinations (300 mg CPZ + ECT and 500 mg CPZ + ECT) did not offer any remarkable therapeutic advantage over 500 mg of chlorpromazine except at week 1, when the 500 mg CPZ + ECT group was better than all the other three treatment groups
Natani *et al*. 1983[44]	3	N = 90; ICD-9 Schiz Comparative trial RCT, RP, PDRS, CDCS	HPL 15 vs. ECT vs. HPL 15 + ECT	All three treatment methods effective in management of schizophrenia In terms of reduction in total scores on RP and PDRS, subjects who received ECT + HPL were significantly better after 1st and 2nd week, but the difference was not significant after 3^rd^ week Similar trends were observed in areas of ‘general appearance and behavior,’ ‘thought and thought processes’ and ‘affect and mood’
Gangadhar[45]	N = 35; Functional Psychosis	Neuroleptic vs. Neuroleptic +ECT		Subjects who had received ≥2 ECTs had less EPS as compared to those not receiving ECT
Bagadia *et al*.[46]	3	N = 78; DBRCT	Real ECT + PBO vs. Simulated ECT + CPZ 300‑900	Similar improvement observed in both the groups on total BPRS score, various BPRS factor scores and CGI
Agarwal and Winny[47]	4	N = 58; DBCT, BPRS, Three phases 1- CPZ only 4 weeks-non responders-received additional ECTfollow-up-4	CPZ 600-1200 + Simulated ECT vs. CPZ + ECT	At the end of first four weeks (Part-1 of the trial), 28 of the 58 subjects showed a reduction of more than 50% in their BPRS score, rest (30 subjects) were assigned to either ECT or simulated ECT Patients in both groups improved significantly No difference between the ECT and simulated ECT groups in response rate during the treatment phase or at follow-up of 1 month after stopping ECT Subject receiving ECT showed significantly better improvement on BPRS score of depression at the end of follow-up. There was no difference in any other domain
Abraham and Kulhara[48]	26	N = 22; RDC Schiz, DBRCT BPRS, CGIS	TFP + Simulated ECT vs. TFP + ECT	The ECT group showed more rapid improvement than the simulated ECT group, but by 12th week scores for both the groups were similar and did not show any significant difference thereafter till 26^th^ week
Sarkar *et al*.[49]	6 months	N = 30; DBRCT	HPL 15 mg + true ECT vs. HPL + sham ECT	Efficacy similar in both groups
Goswami *et al*.[50]		N = 31; DSM IV, DBRCT; TRS, BPRS, CGI	CPZ + Sham ECT vs. CPZ + ECT	Significant decrease in BPRS score in the ECT group from 2^nd^ week onwards (after 6 ECTs) and no significant decrease in the sham ECT group No difference between the two groups in CGI score, need for rescue medications and total daily dose of AP Relatives favored ECT treatment for greater comfort and satisfaction Re-hospitalization rates were less in the ECT group

CPZ - Chlorpromazine; ECT - Electroconvulsive therapy; TRZ - Thioridazine; IMN - Imipramine; Schiz - Schizophrenia; IST - Insulin subcoma therapy; TFP - Trifluoperazine; TFD - Trifluperidol; FFX - Fluphenthixol; SBRCT - Single blind randomized controlled trial; RP - Rockland and polin scale; CGI-S - Clinical global impression severity; RDC - Research diagnostic criteria; BPRS - Brief psychiatric rating scale; ESRS - Extrapyramidal symptoms rating scale; CGI - Clinical global impression scale; RCT - Randomised controlled trial; PDRS - Psychiatric disability rating scale; CDCS - Composite diagnostic checklist of schizophrenia; HPL - Haloperidol; EPS - Extrapyramidal symptoms; DBRCT - Double blind randomized controlled trial; PBO - Placebo; DBCT - Double blind controlled trial; TFP - Trifluoperazine; TRS - Treatment resistant schizophrenia

### Efficacy of centbutindole [Table 4]

Centbutindole was developed by Central Drug Research Institute, Lucknow; it has been evaluated in four trials and has been compared with trifluperazine, haloperidol and risperidone (see Table 5 for this trial).[51,52,53,54] These studies have shown that centbutindole is as efficacious as haloperidol, trifluperazine and risperidone in the treatment of schizophrenia.

**Table 4:** Efficacy studies of centbutindole in schizophrenia

Authors	Duration (in weeks)	Sample size/scale/design	Medication with dose (s) in mg	Outcome	Side effects
Doongaji *et al*.[51]	5 4-CBD 1 - PBO	N = 27; RDC, BPRS, CGI, AIMS, Open trial	8 4-CBD 1 - PBO	Significant reduction in BPRS and CGI scores	S/E noted were tremors, insomnia, dryness of mouth, increased salivation, rigidity, weight gain, drowsiness, akathisia, sleep disturbance, oculogyric crisis, constipation
Doongaji *et al*.[52]	6	N = 40; RDC, BPRS, CGI, ESRS, SESC, DBRCT	CBD-3-4.5 TFP-15-22.5	50% reduction in BPRS in CBD group compared to 44% in the TFP group No significant difference in the total or individual variable BPRS score between CBD and TFP No improvement in the variable of guilt feelings, mannerisms and posturing, grandiosity, depressive mood, hostility, uncooperativeness, excitement, elated mood and motor hyperactivity Significant improvement in the CGI scores in both the groups	S/E noted were EPS and autonomic S/E
Singh *et al*.[53]	6	N = 44; ICD-10 Schizophrenia, PANSS, CGI, UKU	HPL-15; vs. CBD -4.5	CBD had early onset of therapeutic effect for both positive and negative symptoms Both the drugs had comparable efficacy from 3^rd^ week onwards	Comparable or less with CBD

CBD - Centbutindole; PBO - Placebo; RDC - Research and diagnostic criteria; BPRS - Brief psychiatric rating scale; CGI - Clinical global impression scale; AIMS - Abnormal involuntary movement scale; S/E - Side effects; ESRS - Extrapyramidal symptoms rating scale; SESC - Side-Effect symptom check-list; DBRCT - Double blind randomized controlled trial; PANSS - Positive and negative syndrome scale for schizophrenia; HPL - Haloperidol; UKU - Udvalg for kliniske undersogelser side effect rating scale

### Efficacy studies of second generation antipsychotics in schizophrenia [Table 5]

Twenty studies have been published on evaluation of the efficacy/effectiveness of second generation antipsychotics in schizophrenia.[54–73] Of these trials, 11 have reported the efficacy of risperidone, five of clozapine, two of olanzapine, two of aripiprazole and one of quetiapine. Some of these studies have also evaluated different doses of the same medications and some others have evaluated the dosing schedule. All these trials have included subjects diagnosed as schizophrenia on the basis of DSM-IV or ICD-10 except for Agashe *et al*.[58] who used DSM-IIIR criteria. All these trials have used standard instruments to assess the efficacy and side effects. The sample size has varied from 30 to 606 and the duration of these trials have been six weeks to four months except for one trial which evaluated the outcome on risperidone at one year[61] and two studies, which followed up subjects on clozapine for 20 months and three years respectively.[66,69] All the studies on risperidone have shown that it is efficacious in short term. Studies which have compared risperidone with other antipsychotic have shown it to be more efficacious than quetiapine but as efficacious as haloperidol and centbutindole. Agarwal and Chadda[64] demonstrated that there was no difference in efficacy of once daily dose versus twice daily dose of risperidone. The study which followed up the subjects on risperidone for one year showed that compared to haloperidol, more subjects on risperidone had better social functioning, productivity and education and significantly fewer patients had suicidal ideation or attempts and needed rehospitalization.[61] Srivastava *et al*.[62] showed that half of the schizophrenia subjects in India require 3-4 mg/day of risperidone and another one-third improve with dose ranging from 1-2 mg/ day. All the studies which have evaluated clozapine have done so in TRS cases and have reported it to be useful in both short and long term. In the study by Avasthi *et al*.[65] olanzapine was found to be as efficacious as haloperidol on PANSS. Additionally olanzapine was found to be better than haloperidol in reducing associated depressive and anxiety symptoms. Sarin *et al*.[68] reported no difference in efficacy of 10 and 15 mg/day of aripiprazole.

**Table 5:** Efficacy studies of second generation antipsychotics in schizophrenia

Authors	Duration (in weeks)	Sample size/scale/design	Medication with dose (s) in mg	Outcome	Side effects
Agarwal *et al*.[55]	16	N = 29; ICD-10 Schiz, PANSS, BPRS, CGI, side effect scale/open trial Resistant to two of HPL, TFP, CPZ in ≥ 1000 mg CPZ equivalents	Clz 300-450	On BPRS Significant reduction in scores at 9 and 16 week At 9 weeks, 17 (68%) patients had >25% improvement At 16 weeks, 19 (76%) patients had improvement between 26-50% and 6 (24%) had >25% improvement None of the patients improved beyond 50% On PANSS Significant reduction in positive, negative and general psychopathology subscale at 9 and 16 weeks	Tachycardia, hypersalivation and sedation
Agarwal *et al*.[56]	6	N = 177 ; DSMIV Schiz, PANSS, CGI, UKU, open trial	Risp 6-8	Significant improvement on PANSS total, positive, negative and general psychopathology subscale and CGI in 71 out of 146 patients at 6 mg/day In 57 out of 71 patients there was significant improvement after dose was increased to 8mg/day Improvement was seen in patients who had previously not responded to HPL, TFP, FFP, CPZ and PMZ Concentration difficulties, depression, reduced duration of sleep, tension/inner unrest, emotional indifference, diminished sexual desire and headache improved with risperidone therapy in a number of patients	S/E increased from 65.7% at baseline to 72.7% at end of therapy S/E noted were EPS, salivation, postural hypotension There was no serious S/E
Bajaj *et al*.[57]	16	N = 30; DSM-IV Schiz. (predominant negative symptoms), Open label, PANSS	Risp 2-10	Significant improvement in the negative symptoms in all patients Significant improvement in all subscales of PANSS in 23 patients Emergence of positive symptoms in 7 patients (suspiciousness, hostility, delusions and conceptual disorganization, auditory hallucinations)	Not reported in study
Agashe *et al*.[58]	4 month	N = 30; DSM IIIR (Schiz and Schizophreniform psychosis), Open label, PANSS, BPRS, CGI, ESRS	Risp 6-16	18 patients (66.7%) showed improvement on PANSS 17 patients (63%) showed improvement on BPRS 17 patients (63%) were considered as much improved and 18 (66.7%) were considered not ill on CGI	No significant difference was noted on the ESRS scores Nausea, vomiting, tremors and weight gain were noted
Desai *et al*.[59]	3 months	N = 28; DSM-IV Schiz, TRS, Open trial, BPRS	Clz 225-325	The BPRS scores showed significant decrease at 1 and 3 months compared to the base line At three months 11 patients showed >50% reduction on BPRS, 14 patients showed between 26-50% reduction on BPRS and only three patients had shown < 25% improvement	Sedation, sialorrhoea, hypotension, weight gain None of the patients developed agranulocytosis
Chaudhuri *et al*.[60]	4	N = 35; ICD-10 ATP, SBRCT, BPRS, GAF	Risp-4 HPL-15	No significant difference in lowering of BPRS score or CGIS scores in the two groups. Subjects in both the groups had significant reduction in BPRS at 4 weeks compared to baseline rating.	All patients in HPL group and 40% patients in Risp group required THF More subjects in the Risp group required lorazepam (for sedation), propranolol (for akathisia) and parenteral diazepam as compared to HPL group
Shrivastava and Gopa[61]	1 year	N = 100; DSMIV, PANSS, CGI, ROT	Risp 2 Vs. HPL 5-15	Significantly more reduction (68.4%) in general psychopathology subscale in haloperidol group as compared to risperidone group while there was no significant difference in other two sub groups Significantly more number of patients showed improvement on CGI in the risperidone group Risp group showed significantly more patients improving in social functioning, productivity and education Significantly fewer patients in Risp group had suicidal ideation or attempts and needed rehospitalization No significant difference between two groups in economic independence and exacerbation	Not reported in study
Srivastava *et al*.[62]	3 months	N = 606; DSMIV, PANSS, post marketing survey	Risp Three groups 1-2mg 3-4mg ≥5mg	Duration of illness was significantly less in the 1-2 mg/day group After three months, 54.5% of the patients were maintained on 3-4 mg/day, 35.5% on 1-2 mg/day and 10% on ≥ 5 mg/day Significant improvement in all the three groups; clinically as well as on PANSS total and PANSS subscores	S/E noted in 14.5% of the patients Akathesia was the most common S/E There was no significant difference in the S/E in the three groups
Suresh Kumar *et al*.[63]	12	N = 24; DSMIV, Open trial, PANSS, CGI, SAS	Risp 2-8	Significant reduction in the PANSS positive, negative, general psychopathology and depression subscale at 2, 4, 8, 10 and 12 weeks Significant improvement in CGI score at 4, 8 and 12 weeks	50% of the patients showed moderate to severe S/E S/E noted were tremor, rigidity, gait disturbances, salivation, akathisia and dystonia
Agarwal and Chadda[64]	8	N = 44; ICD-10 Schiz, PANSS, CGI, DOTES/ROT, OD Vs. BD	Risp 4-8 OD Vs. BD dosing	82% of OD and 79% of BD dose subjects showed a significant treatment response on PANSS No significant differences were observed between the two groups in response pattern on PANSS total or subscale score No significant difference between the two groups on CGI	14 (61%) patients in OD group and 13 (68%) in the twice daily group reported S/E EPS, insomnia and anxiety were the commonest S/E 2 OD group patients discontinued the medication because of S/E while 1 BD group patient discontinued medication because of adverse effects Non significant change in blood pressure and heart rate were observed in both groups
Avasthi *et al*.[65]	12	N = 30; DSM-IV Schiz, ROT, CGI, BPRS, PANSS, SANS, MADRS, HAM-A, QOL, UKU, SAS, BARS	Olz 5-20 vs. HPL5-20	Significant improvement in the total PANSS, PANSS positive, negative and general psychopathology subscale, total BPRS, BPRS positive score, CGI severity scale in both groups No significant improvement in the BPRS- negative and BPRS- anxiety scale in HPL group but there was significant improvement in the Olz group On MADRS and HAM-A, HPL group showed no significant improvement while, Olz group showed significant improvement on both scales Olz group showed significant improvement in avolition - Apathy, anhedonia and attention subscales and SANS total score, but not on others HPL group showed no improvement in either of the subscale or total SANS score	Both groups did not report any epileptic seizures, EPS or any side effects related to CVS, GI, dermatology, sexual and reproductive functioning Weight gain, sleepiness and increased sleep duration and asthenia was more common in the Olz group There was no significant change in the BARS, SAS and QOL scale in either group Rigidity, tremors and tension/inner unrest was more commonly reported by HPL group
Srivastava *et al*.[66]	3 years	N = 25; TRS Open label, BPRS, PANSS	Clz 248.21	Significant reduction in psychopathology on BPRS and PANSS was observed in 85% of patients An improvement in social functioning was evident, with seven patients pursuing a career independently, and another six working with their family members since being started on clozapine No patient required hospitalization	There was no incidence of granulocytopenia Sedation, sialorrhea and weight gains were the most frequently observed unwanted effects
Chandra *et al*.[54]	8	N = 44; ICD-10 DCR Schizophrenia DBRCT, PANSS, CGI, UKU	CBD-4.5 vs. Risp-6	CBD and Risp have similar onset of action Significant reduction in the total PANSS score, positive, negative and general psychopathology score from 2^nd^ week onwards in both groups Steady and significant decline in the score from 2^nd^ week onwards CBD had similar improvement on CGI as Risp	S/E were comparable between CBD and Risp except 5 patients developed dystonia in CBD group as compared to 1 in Risp group Weight gain, increased salivation, tremors, lassitude, sedation, concentration difficulties, rigidity, increased sleep, hypokinesia, orthostatic dizziness, accommodation disturbance, constipation, increased sweating, inner unrest, amenorrhea, depression, akathisia
Chavda *et al*.[67]	4	N = 249; DSMIV Schiz. Or Schizoaffective ROT, PANSS, SAS, AIMS, Multicentric	Arip 15	Significant reduction in PANSS total and all subscale score of PANSS which was seen from 2^nd^ week onwards	S/Es were mild to moderate in intensity and not required drug discontinuation
Sarin *et al*.[68]	6	N = 136; ICD-10 Schizophrenia, ROT, Multicentric	Arip 10 vs. Arip 15	Aripiprazole treatment, in the dose of 10 and 15 mg consistently decreased the total PANSS score over the study duration. The decrease in PANSS score (compared to baseline) reached statistical significance (P < 0.001) from week-2 No significant difference between the 2 doses in terms of efficacy	The most commonly reported adverse events were fatigue (32%), insomnia (27%), headache (24%), nausea/vomiting (19%), tremor (20%), rigidity (5%), akathisia (5%), constipation (12%) There was no significant difference between the two doses in terms of S/Es
Raguraman *et al*.[69]	20 months	N = 22; Open label, TRS, PANSS, GAF, CDS, AIMS	Clz 300-400	50% of the patients responded to Clz Significant decrease in PANSS negative and general psychopathology score There was decrease in suicidal ideas Improvement in global functioning, AIMS score Patients with paranoid schizophrenia showed a better response compared to undifferentiated schizophrenia	
Vijay Sagar and Chandrashekar[70]	6	N = 46; DBRCT, Paranoid Schiz. PANSS, CGI, UKU	Risp 1.39 vs. HPL 1.7	Significant improvement in psychopathology on PANSS in both the groups but there was no difference between the two drugs in the improvement of positive and negative symptoms Risperidone was better than haloperidol in improving the general psychopathology as well as in bringing about global improvement	Significant difference between the 2 groups in terms of sedation, increased duration of sleep, tremor, constipation, polyuria/polydipsia and weight gain
Potkin *et al*.[71]	2 week-mono therapy 4 week-additive therapy	N = 382; DSMIV Schiz, Acute Exacerbation MINI-Plus, PANSS, CGI, HDRS, RDQ, MSQ, SAS, BARS, AIMS/DBRCT Multicentric (12 centres in India)	Risp 4-6 vs. Quet 400-800 vs. PBO	Significant decrease in PANSS total score, CGI-C in Risp group as compared to quetiapine or PBO during the monotherapy treatment No significant difference in decrease in PANSS score between quetiapine vs. PBO or combined atypical (Risp + quetiapine) vs. PBO Significant decrease in HDRS score in Risp group compared to PBO group but not quetiapine Significantly high response rate on PANSS was observed in Risp group as compared to quetiapine or PBO RDQ and MSQ scores were significantly higher in Risp group compared to other two groups No significant difference between quetiapine or PBO group during monotherapy phase Significantly more patients in the quetiapine (53%) and placebo (59%) groups received additional psychotropics (mainly antipsychotics), there was no significant difference in their use Total PANSS score improvement was significantly greater for Risp as compared to PBO but not quetiapine Significant decrease in CGI-C and CGI-S decease in Risp group as compared to quetiapine or PBO	There was no difference in the percentage of patients reporting side effects Insomnia and headache were the most common side effects In addition, cogwheel rigidity and akathisia were reported in Risp group, while Quet group reported dizziness, somnolence, sedation and constipation
Thomas *et al*.[72]	6	N = 130 + 100 controls; DSM-IV DIGS, CGI-C, CGI-S, AIMS, SAS, Open label	Olz 5-30	At 6 weeks, there was a significant improvement in PANSS total, PANSS positive, negative, general psychopathology, CGI-S and CGI-I scores There was significant improvement in the efficacy index At 6 week, 72 (55%) of the patients responded on PANSS Average drug dose was higher among the non-responders	Responders had lower levels of insulin, SAS score and AIMS score Significantincrease in weight Significant decrease in EPS score No significant change in the systolic or diastolic blood pressure No significant association was found between SAS and AIMS score or changes in insulin levels with tested genetic markers or polymorphism
Dutt *et al*. (in press)[73]	4 years	N = 51; Retrospective study; about two-third TRS	Clz 125-600	During the inpatient stay (mean duration 63 days), there was significant reduction (34.7%) in total PANSS rating after starting clozapine The mean duration of follow-up was 3.99 (SD 3.13) years after starting clozapine. Of the 51, 45 cases were on clozapine at the time of last follow-up and in 6 cases clozapine was stopped due to high cost (N = 2), seizure (N = 1), leucopenia (N = 2) and poor response (N = 1) Clozapine led to reduction in suicide attempts,	Sialorrhea (58.8%) was the most common side effect reported Other side effects reported were sedation (47.0%), constipation (15.6%), severe hypotension (2%), urinary incontinence (2%) and urinary retention (2%) Three cases (5.88%) developed leucopenia Four patients (7.8%) developed seizures

Schiz - Schizophrenia; TRS - Treatment resistant schizophrenia; PANSS - Positive and negative syndrome scale for schizophrenia; BPRS - Brief psychiatric rating scale; CGI - clinical global impression scale; HPL - Haloperidol; TFP - Trifluoperazine; CPZ - chlorpromazine; Clz - Clozapine; UKU - Udvalg for kliniske undersogelser side effect rating scale; Risp - Risperidone; FFP - Fluphenazine; PMZ - Pimozide; ATP - Acute and transient psychotic disorder; SBRCT - Single blind randomized controlled trial; GAF - Global assessment of functioning; THF - Trihexyphenidyl; SAS - Simpson angus rating scale for extrapyramidal side effects; DOTES - Dosage record and treatment emergent symptom scale; OD - Once a day; BD - Twice a day; Olz - Olanzapine; CVs. - Cardiovascular system; GI - Gastrointestinal; SANS - Scale for assessment of negative symptoms; MADRS - Montgomery - Asberg depression rating scale; QOL - Quality of life scale; HAM-A - Hamilton’s anxiety rating scale; BARS - Barnes akathisia rating scale; DCR - Diagnostic criteria for research; ROT - Randomized open trial; DBRCT - Double blind randomized controlled trial; CBD - Centbutindole; Arip- Aripiprazole; CDS - Calgary depression scale; MINI-Plus- Mini international neuropsychiatric interview—Plus version; HDRS - Hamilton rating scale for depression; RDQ - Readiness for discharge questionnaire; MSQ - Medication satisfaction questionnaire; Quet- Quetiapine; DIGS - Diagnostic interview for genetic studies; CGI-S - Clinical global impression severity; CGI-C - Clinical global impression -Change; PBO - Placebo; AIMS - Abnormal involuntary movement scale; S/E - Side effects; ESRS - Extrapyramidal symptoms rating scale

### Efficacy of antipsychotics in other disorders: mania [Table 6]

Some of the older studies included a few subjects with mania. Three studies in the early 60s included very few subjects in the open label design studies and reported efficacy of haloperidol, thioridazine and thioproperazine in the treatment of mania.[4,6,8] One study included only one case of treatment refractory mania and showed that oral fluphenazine was not useful in such subjects.[5] Another study evaluated the efficacy of zuclopenthixol in management of 25 cases of mania. This study did not specifically report the efficacy of zuclopenthixol in mania, but irrespective of the diagnosis the reported outcome suggests that zuclopenthixol for eight weeks was useful.[74]

**Table 6:** Efficacy studies of antipsychotics in mania

Authors	Duration (in weeks)	Sample size/scale/design	Medication (s)	Dose (s) in mg	Outcome	Side effects
Damania and Masani[4]	8 days-4months	N = 19	TRZ	30-300	Of 19 subjects with MDP, 14 improved of which 6 were cured	Drowsiness and giddiness No serious S/E were noted
Bhaskaran, 1963[6]	6	N = 4 ; Open label	TPZ	60	All 4 subjects with mania recovered	
Jetley[8]	6	N = 6 Open label	HPL	1.5-4.5	All 6 subjects of mania showed improvement	
Fernandes*et al*.[74]	8	N = 25; CGI, BPRS, UKU, open study	Zuclopenthixol	Zuclopenthixol acuphase-50-150 Zuclopenthixol decanoate 200-600	Zuclopenthixol Acetate (acuphase) -category of severely ill reduced from 55% to 20.8% at 72 hours CGI very much improved and much improved - 27% at 24 hours and 34% at 72 hours Zuclopenthixol decanoate (depot)-CGI “very much Improved” 10% of the cases at the baseline, in 24% at 4 weeks and in 35% at 8 weeks. BPRS scores also showed significant reduction from a mean total score of 58 at the baseline to 39 at 4 weeks and 36 at 8 weeks	Neurological S/E (tremors, rigidity, hypokinesia, dystonia, akathesia, hyperkinesia), depression, asthenia, increased salivation, sedation, hypotension, headache
Khanna *et al*.[75]	3	N = 291 DSM-IV, DBRPCT, YMRS, CGI, GAS, MADRS, PANSS, ESRS/Multicentric	Risp vs. PBO	1-6	Significantly higher reduction in YMRS score at 1st, 2nd and 3rd week in Risp group compared to PBO >50% reduction in YMRS score was seen in 73% patients in Risp group compared to 36% in PBO group Significant improvement in Risp group in CGI, GAS scores, PANSS total, MADRS Patients with mania had significantly greater improvement in YMRS score There was significant improvement in Risp group irrespective of age, gender and baseline YMRS score	35% of subjects in Risp group experienced EPS (compared to 6% in the placebo group) Other side effect which was reported by more than 10% of subjects was insomnia; however, there was no difference between the 2 groups on the incidence of the same
Smulevich *et al*.[76]	12	N = 438 DSM IV, DBRPCT YMRS, CGI, UKU	HPL Vs. Risp Vs. PBO	Risp 2-6 HPL 4-12	Significantly greater improvements in mean YMRS scores were seen with risperidone than with placebo at week 1 and at each subsequent assessment during the first three weeks. Differences between risperidone and haloperidol were not significant At the three weeks, more patients were treatment responders (>50% reduction in YMRS total scores) among those treated with risperidone (48%) or haloperidol (47%) than placebo (33%). The difference between risperidone and placebo was significant (*P* < 0.01) At 12 weeks, a > 50% reduction in YMRS total score was maintained in 98% (53/54) and 100% (37/37) of patients who responded at 3 weeks and continued on double blind therapy with risperidone and haloperidol, respectively. An additional 83% (30/36) and 89% (24/27) of subjects who did not reach a reduction of .50% in the YMRS total score on risperidone or haloperidol, respectively, at 3 weeks but continued on double-blind therapy were responders at 12 weeks	Severity of most adverse events was mild or moderate Adverse events reported in $ 10% of patients during 12 weeks of doubleblind treatment were extrapyramidal disorder (24%), somnolence (10%), and hyperkinesia (10%) in the risperidone group and extrapyramidal disorder (43%), hyperkinesia (19%), tremor (13%), and hypertonia (10%) in the haloperidol group Mild or moderate depression was reported in seven (5%) risperidone patients and eight (6%) haloperidol patients

TRZ - Thioridazine; MDP - Manic depressive psychosis; S/E- Side effect; HPL - Haloperidol; TPZ - Thioproperazine; CGI - Clinical global impression scale; BPRS - Brief psychiatric rating scale; UKU - Udvalg for kliniske undersogelser side effect rating scale; DBRPCT - Double blind randomized placebo controlled trial; YMRS - Young mania rating scale; GAS - Global assessment scale; MADRS - Montgomery - Asberg depression rating scale; PANSS - Positive and negative syndrome scale for schizophrenia; ESRS - Extrapyramidal symptoms rating scale; Risp - Risperidone; PBO - Placebo; EPS - Extrapyramidal symptoms

However, in the recent trials, atypical antipsychotics have been evaluated in large samples in double blind placebo control trials and the outcomes have been measured using standard instruments. In the study by Khanna *et al*.[75] risperidone was found to be significantly better than placebo at weeks 1 and 2 and 3 (total YMRS: *P* < 0.01). Another multicentric trial, which included subjects from India too, showed that both risperidone and haloperidol were better than placebo; however, there was no difference in the efficacy of risperidone and haloperidol.[76] At three weeks, more patients were treatment responders (>50% reduction in YMRS total scores) among those treated with risperidone (48%) or haloperidol (47%) than placebo (33%). The difference between risperidone and placebo was significant (*P* < 0.01). At 12 weeks, the response rate was maintained in almost all subjects who responded at three weeks and additionally more than 80% of the subjects who did not respond (reduction of >50% in the YMRS total score) to risperidone or haloperidol at three weeks but continued the medications responded at 12 weeks.

### Acute psychosis [Table 7]

Four studies have reported the efficacy of antipsychotics in the management of acute psychosis, of which three have specified that the trials included subjects with acute and transient psychosis,[60,74,77] while another trial mentioned the diagnostic category only as acute psychosis.[78] Trial by Agarwal and Sitholey[77] involved children and adolescents presenting with acute and transient psychosis and is discussed later. These studies suggest that thioproperazine is better than chlorpromazine and there is no difference in short term efficacy of risperidone and haloperidol in acute and transient psychosis. As mentioned earlier Fernandes *et al*.[74] did not specifically report the outcome in acute and transient psychosis, however, irrespective of the diagnosis the reported outcome suggests that zuclopenthixol for eight weeks was useful.

**Table 7:** Efficacy studies of antipsychotic in acute psychosis

Authors	Duration (weeks)	Sample size/scale/design	Medication (s)	Dose (s) mg	Outcome	Side effects
Mokashi and Chandorkar[78]	5 days	N = 60; DBPCT, Consecutive sampling, Acute psychosis	TPZ CPZ vs. PBO	TPZ 15 CPZ 50	In the TPZ group, 17 subjects had relief of symptoms, 1 showed good improvement, 1 partial improvement and 1 no improvement In the CPZ group, 8 subjects had relief of symptoms, 2 showed good improvement; 7 showed partial improvement and 3 no improvement In the placebo group, 4 subjects had relief of symptoms, 1 showed good improvement, 2 partial improvement and 13 no improvement TPZ was more effective than CPZ in overactivity, agitation and over talkativeness Male subjects showed better response than female subjects	The hypokinetic hypertonic reaction was noted in 8 cases receiving TPZ and in 4 cases receiving CPZ group All cases in both groups responded to phenargan Hypotension was noted in one case under CPZ group Placebo did not show any side reaction In none of the cases the trial had to be abandoned due to toxic reaction
Fernandes *et al*.[74]	8	N = 46; ATP, BPRS	Inj Zuclo phenthixol	Zuclo penthixol acetate-50-250 Zuclo penthixol-200-600	Zuclopenthixol Acetate (acuphase) -category of severely ill reduced from 55% to 20.8% at 72 hours Mildly ill category increased from 0.8% to 15% during the same period CGI very much improved and much improved - 27% at 24 hours and 34% at 72 hours Zuclopenthixol Decanoate (depot)-CGI “very much Improved- 10% of the cases at the baseline, in 24% at 4 weeks and in 35% at 8 weeks BPRS scores also showed significant reduction from a mean total score of 58 at the baseline to 39 at 4 weeks and 36 at 8 weeks	Neurological S/E (tremors, rigidity, hypokinesia, dystonia, akathesia, hyperkinesia), depression, asthenia, increased salivation, sedation, hypotension, headache.
Chaudhuri *et al*.[60]	4	N = 35; ICD-10 ATP, SBRCT, BPRS, GAF	Risp vs. HPL	Risp-4 HPL-15	There was no significant difference in lowering of BPRS score in the two groups. Subjects in both the groups had significant reduction in BPRS at 4 weeks compared to baseline rating.	All patients in HPL group and 40% patients in Risp group required THF More subjects in the Risp group required lorazepam (for sedation), propranolol (for akathisia) and parenteral diazepam as compared to HPL group

DBPCT - Double blind placebo controlled trial; TPZ - Thioproperazine; CPZ - Chlorpromazine; PBO - Placebo; ATP - Acute and transient psychotic disorder; CGI - clinical global impression scale; BPRS - Brief psychiatric rating scale; SBRCT - Single blind randomized controlled trial; GAF - Global assessment of functioning; Risp - Risperidone; HPL- Haloperidol; THF- Trihexyphenidyl

### Anxiety

Studies in 1970s and 1980s evaluated the efficacy of low dose haloperidol,[79] flupenthixol,[80] trifluperidol,[81] pimozide,[82] prochlorperazine,[83] trifluperazine[84] in various anxiety states and reported these to be efficacious. The study which used pimozide compared it with chlordiazepoxide and reported it to be useful, but inferior to chlordiazepoxide.[82] The study which compared trifluperazine and chlordiazepoxide combination with prochlorperazine reported the latter to be better.[83]

### Agitation and violence

Two studies have evaluated the role of antipsychotic in violent and agitated subjects attending the psychiatry emergency settings. In a randomized controlled trial, Alexander *et al*.[84] compared the efficacy of injectable lorazepam and a combination of injectable haloperidol and promethazine and reported that after four hours of the injection, 96% of the subjects in both the groups were tranquil or asleep. However, in terms of being asleep, 76% of the subjects given haloperidol-promethazine were asleep compared to 45% of subjects in the lorazepam group. The haloperidol-promethazine combination also produced a faster onset of tranquillization/sedation and more clinical improvement over the first 2 h. Neither intervention differed significantly in the need for additional intervention or physical restraints, subjects absconding, or adverse effects. In another randomized study from the same centre, Raveendran *et al*.[85] compared the efficacy of intramuscular olanzapine with intramuscular haloperidol plus promethazine in 300 subjects and reported that both treatments led to similar proportions of subjects being tranquilized or asleep at 15 minutes (olanzapine-87%, haloperidol plus promethazine-91%) and 240 minutes (olanzapine-96% and haloperidol plus promethazine-97%). However, compared to those given haloperidol plus promethazine, more subjects in the olanzapine group required additional drugs over four hours (21% vs. 43%). Adverse effects were uncommon with both treatments.

### Delirium

Only a small case series of seven cases reported that risperidone is effective in the management of delirium and it is well tolerated by these subjects.[86]

### Delusional disorders

Good response to antipsychotics like trifluperazine, haloperidol and chlorpromazine was reported in cases of delusional parasitosis.[87] In their study on delusional disorder, Grover *et al*.[88] found that best response was seen with risperidone, followed closely by pimozide. Of the 20 subjects treated with risperidone, six were considered to have good response (>75% reduction in symptoms) and nine were considered to have partial response (>25 but <75% reduction in symptoms). Of the 18 subjects treated with pimozide, four qualified for good response and six as partial responders.

### Use of Antipsychotic in Children and Adolescents [Table 8]

Seven studies have evaluated the efficacy of various antipsychotics in childhood disorders or childhood/adolescent onset disorders. Studies done in 1960s and early 70s evaluated the role of antipsychotics primarily in the behavioral problems in children with epilepsy/epilepsy and mental retardation and have shown that trifluperazine, prothipendyl and trifluperidol are useful but fluphenazine is ineffective. One small study evaluated the usefulness of clozapine in childhood onset schizophrenia.[91] A study which evaluated the role of risperidone in autism showed promising results[92] and another study demonstrated the efficacy of olanzapine in childhood/adolescent onset acute and transient psychosis.[77] However, the studies which used olanzapine and risperidone showed that these drugs lead to reasonable weight gain, which may be cause of concern.

**Table 8:** Efficacy studies of antipsychotic in children and adolescents

Authors	Duration (in weeks)	Sample size/scale/design	Medication(s)	Dose(s) mg	Outcome	Side effects
Doongaji *et al*.[2]	6	N = 12; Mental sub normality	Prothipendyl vs. PBO	120-240	3 out of 6 subjects with mental sub normality who received the drug improved significantly in restlessness. No improvement was noted in subjects treated with PBO	Not reported specifically for subjects with mental sub normality
Bassa[89]	4-24	N = 34; Children with weakness in limbs (Cerebral Palsy) -16 also had psychotic features	TFP	1-15	13 cases showed improvement while 21 cases had not improved; subjects with psychotic features had better response Improvement was maintained in 6 cases for 24 weeks and for 2 weeks in the rest of the cases Subjects with psychosis showed maximum improvement	S/Es observed were drooling, anorexia, drowsiness, headache, vomiting, constipation, redness of lips and tongue, swelling of both upper and lower eyelids
Somasundaram[90]	6	N = 11; PCT Chronic Epilepsy Children for behavioural Sx	FFP vs. PBO (in addition to antiepileptics)	1-2 mg	FFP was not found to be effective in controlling the behavioral symptoms in mentally retarded children.	No adverse effects were noted on the course of epilepsy in these children
Bagadia *et al*.[80]	10 weeks	N = 73; (Children with aggression and hyperkinesis in emotional disturbance, epilepsy and MR)	TFD	0.5-2	Children having hyperkinesis and aggressiveness due to emotional disturbances and had normal intelligence responded best; 14/22 (65%) as compared to 10/41 (24%) in epilepsy and/or mental retardation group. One patient with post traumatic behavior problem showed significant improvement	EPS (including tremor), giddiness, weakness, drowsiness, restlessness, anorexia, dryness of mouth, nausea, skin rash
Malhotra *et al*.[91]		N = 5; COS Open label BPRS, CGI	CLZ		Positive symptoms improved more than the negative symptoms There was no change in the ritualistic behavior There was substantial global clinical improvement	No serious S/E (seizures, agranulocytosis) were observed
Nagaraj *et al*.[92]	6 months	N = 40; Children with autism, DBRPCT CARS, CGAS, VSMS	Risp vs. PBO	1-6	12 out of 19 children showed improvement in total CARS score and 17 in CGAS score in Risp group. No child in PBO group showed improvement on CARS and only 2 showed some improvement on CGAS. These differences were statistically significant On parent questionnaire, there was improvement in social responsiveness, nonverbal communication, hyperactivity and aggression in Risp group Parents rated 9 out of 19 children as “improved to some extent” and similar number as “considerably improved” in Risp group; In PBO group 6 out of 20 children were rated to have improved to some extent, 9 were reported to have shown no change and 4 had “worsened” after treatment. These differences were not statistically significant After treatment discontinuation, relapse of disruptive behavior was seen in 9 children within 3 weeks of stopping risperidone	Increased appetite and improved eating habits were reported in 17 children receiving risperidone. The mean weight change in the risperidone group was 2.81 kg (SD = 2.04 kg), an increase of 17%, whereas that in the placebo group was 1.71 kg (SD = 1.3), an increase of 9.3%. These changes were, however, not statistically significant
Agarwal and Sitholey[77]	6	N = 23; Pediatric ATP, Open label, BPRS, CGI, DOTES	OLZ	5-20	There was significant improvement in BPRS and CGI score On CGI, 14 (60.9%) patients were rated as markedly improved, 8 (34.8%) improved and 1 (4.3%) as minimally improved	S/E: Dryness of mouth, increase in appetite, weight gain, drowsiness, headache, dizziness, constipation, blurring of vision, and decreased appetite. No patient developed EPS. Significant weight gain was observed

PBO - Placebo; TFP - Trifluoperazine; S/E - Side effects; PCT - Placebo controlled trial; FFP - Fluphenazine; MR - Mental retardation; TFD - Trifluperidol; EPS-Extrapyramidal symptoms; COS - Childhood onset schizophrenia; BPRS - Brief psychiatric rating scale; CGI - Clinical global impression scale; CLZ - Clozapine; DBRPCT - Double blind randomized placebo controlled trial; CARS - Childhood autism rating scale; CGAS - Children’s global assessment scale; VSMS - Vineland social maturity scale; Risp - Risperidone; ATP - Acute and transient psychotic disorder; DOTES - Dosage record and treatment emergent symptom scale; OLZ - Olanzapine

## RESEARCH ON OTHER TREATMENT RELATED ISSUES

### Pharmacogenomics

With increasing interest in the role of genetic factors in treatment response and side effects of medications, studies from India have tried to address the genetic links of tardive dyskinesia, treatment response and severity of psychopathology.[93–96] However, the findings are preliminary and inconclusive. Tiwari *et al*.[93] evaluated the role of six single nucleotide polymorphisms (SNP) in tardive dyskinesia and showed that CYP1A2 1545 C > T SNP was associated with TD (*P* = 0.03) and schizophrenia (*P* = 0.04), but the association was rendered insignificant after corrections for multiple comparisons. Vijayan *et al*.(2007)[94] studied various alleles, genotypes, haplotypes and their linkage disequilibrium and observed that H313HTT genotype was associated with schizophrenia and TaqIB1B1 genotype was significantly associated with higher psychopathology score. Subjects with H313HCC, TaqIA2A2 and Taq1D1D1 had higher mean improvement scores. Distinct shift in the linkage disequilibrium pattern of responder and non responder group was observed. Thomas *et al*.[95] reported that average olanzapine dose, baseline weight and dopamine receptor D-4 (DRD4-120 bp) duplication marker have significant association with the efficacy index. Gupta *et al*.[96] reported significant allelic associations of two SNPs (rs4633 and rs4680) with drug response.

### Predictors of Treatment Response

A study reported that subjects, who showed initial dysphoric response to a test dose of neuroleptic, later respond poorly to the neuroleptics.[97]

### Prescription Patterns

A few studies have evaluated the antipsychotic prescription patterns from India. In one of the early studies, Khanna *et al*.[98] evaluated the psychotropics drug prescription pattern in chronic long stay patients at Ranchi and compared the prescription trends in 1984 and 1988. Most of the patients whose prescriptions were reviewed were suffering from schizophrenia. More than one antipsychotic medication was prescribed to 13% in 1984, which fell down to 7% in 1988. Further it was seen that very few patients received anticholinergic agents and the use of benzodiazepines increased over the years (4% in 1984 and 10% in 1988), which authors attributed to development of distressing tardive dyskinesias over the years. In an evaluation of prescriptions at discharge of patients with schizophrenia, Padmini Devi *et al*.[99] reported that risperidone was the most commonly prescribed antipsychotic (56.17%), followed by olanzapine (21.34%) and quetiapine (3.93%). Typical antipsychotics were used only in 15.73% of cases and polypharmacy (concurrent use of more than 1 antipsychotic) was seen in 9% of cases. In another study from Jammu, Shanwey *et al*.[100] evaluated the prescription of 270 outpatients, and reported that fixed dose formulation of trifluperazine, chlorpromazine and trihexyphenidyl (parkinforte) was used in 45.4% of cases followed by chlorpromazine[36.3%] and quetiapine [34.5%]. The authors also found that typical antipsychotics were used in 82.72% of cases and polypharmacy was seen in 72.72% of cases. Dutta *et al*.[101] evaluated the prescription patterns in 118 stable schizophrenia subjects and reported that on an average 2.8 medications were prescribed to each subject and olanzapine was the most commonly prescribed antipsychotic followed by haloperidol and risperidone. About half of the subjects were receiving more than one antipsychotic. All these findings suggest that antipsychotic prescriptions vary from centre to centre and possibly have changed over the years.

In a recently conducted survey, Grover and Avasthi[102] found that the three most commonly prescribed antipsychotics by Indian psychiatrists were risperidone, olanzapine and haloperidol. Typical antipsychotics comprise 25.15% of all prescriptions and in about 22.36% of cases the psychiatrists were using more than one antipsychotic in the same patient. Another survey specifically assessed the prescribing practices of clozapine; only 28% of psychiatrists reported that their prescription of clozapine was guided by their knowledge about the efficacy of clozapine. Majority of the psychiatrists opined that clozapine leads to symptom reduction to the extent of 40 to 70%, and the average dose required for stabilization was between 75 to 300 mg/day. Only 16% of psychiatrists preferred to combine clozapine with other antipsychotics. In terms of blood monitoring, 80% of the psychiatrists monitored the blood counts weekly in the first month of therapy and then once monthly for next 6 months and further monitoring was done as per the need.[103]

### Treatment continuation, compliance and attitude towards treatment

Khanna *et al*.[104] reported that 31% of the subjects with schizophrenia do not keep their appointment for detail evaluation after initial evaluation in the walk-in clinic. The authors also reported that 32% of the subjects stop attending the clinic after initial detailed workup and diagnostic clarification. In another study from the same center, Kulhara *et al*.[105] reported that 25% of schizophrenia subjects do not come for follow-up after six months of detailed workup and diagnostic clarification and 23% of subjects with schizophrenia don’t seek any medical help in next five years. Murthy *et al*.[106] studied a mixed group of patients (which included subjects of psychosis also) and reported that duration of illness more than six months, residence at a distance of more than 50 kilometers from the hospital and psychiatric diagnosis other than functional psychosis favored treatment discontinuation. Ponnudurai *et al*.[107] evaluated the treatment adherence in 111 cases of psychotic illness (mostly schizophrenia) by Ferric Chloride reagent test to look for excretion of phenothiazine in urine and reported non-adherence in 19% of subjects. In another interesting study Srinivasan and Thara[108] reported that history of noncompliance with oral medication was seen in about 58% of patients during the course of their illness and half of these subjects were given oral medications at least once without their knowledge by the family members under the psychiatrist’s advice. It was seen that spurious administration of antipsychotic leads to reduction in symptoms in 91% of subjects given medications in this way and helps to convince the patient to take oral medications subsequently. Half of the caregivers who participated in the study felt that spurious administration of antipsychotic was the right action under the circumstances. In a recent study, Baby *et al*.[109] reported rates of non-compliance with medication to be 38.7% in subjects with schizophrenia. The authors also reported that a majority of the patients and family members had a positive attitude towards medication and treatment. Further, family members are able to identify the compliance status of the patients and the reasons for the noncompliance better than the patients. The factors which had significant influence on the medication compliance included perceived daily benefit from medication, positive relationship with the psychiatrist, pressure from the family and health system and positive family belief towards illness and treatment. The significant reasons for noncompliance are no perceived daily benefit from medications, difficulty in gaining access to treatment and medications, financial obstacles, embarrassment or stigma related to treatment and medications and medicines currently not perceived as necessary. Besides attitude, other factors which are significantly associated with noncompliance include lower educational status, rural area of stay, adjustment difficulty with family and spouse, previous history of non-compliance, poor insight into illness, higher positive PANSS score. Past history of hospitalization was associated with better compliance with medications.

### Impact of antipsychotics on disability

A recently published study, which compared three groups of community dwelling subjects with schizophrenia, showed that mean disability scores remain virtually unchanged in those who remained untreated, but showed a significant decline (indicating decrement in disability) in those who continued to receive antipsychotics and in those in whom antipsychotic treatment was initiated. The proportion of patients classified as ‘disabled’ declined significantly in the treated group, but remained the same in the untreated group.[110]

### Costs

A few studies have evaluated the cost of antipsychotics per se and cost of management of schizophrenia. Girish *et al*.[111] found that antipsychotic drugs are affordable and comparable to drug treatment costs of other physical illnesses. They found the monthly cost of treatment with chlorpromazine was Rs.55, an equivalent dose of trifluperazine amounted to Rs.25/month, risperidone Rs.60 and clozapine Rs.225 per month. They also concluded that although antipsychotic drugs are affordable, the other costs associated with treatment make them more expensive, like co-prescribed antiparkinsonian agents, antidepressants, anxiolytics etc. Sarma[112] showed that cost of one outpatient visit was Rs.201 in which medications accounted for less than 10% of the total cost. Grover *et al*.[113] found that the total annual cost of care of schizophrenia amounted to Rs.13687.38, which was similar to cost of treatment of diabetes mellitus. Money spent by patients on buying drugs constituted about 18% of the total costs. A study from Chennai showed that despite the cost of blood tests, the total cost of treatment with clozapine came down by nearly 25% compared to the cost before clozapine was started.[114] In another study, Despande[115] reported that the cost of medication to the hospital which provided free medications to subjects with schizophrenia was Rs.288-less than the cost of medications for bipolar disorders.

### Research on Tolerability and Side Effects of Antipsychotic [Table 9]

Besides the efficacy or usefulness studies, some studies have specifically evaluated the side effects and tolerability issues with antipsychotics.[116–136] In one of the earliest studies, Sarada Menon and Ramachandran[116] reported side effects of subjects who participated in two of their drug trials and had received either trifluperazine or trifluperidol. Other studies have evaluated the rates of skin pigmentation with phenothiazines,[117] rise in prolactin levels with chlorpromazine and trifluperazine[119] and changes in serum electrolytes.[120] Studies have reported prevalence rate of akathesia (25-28%), tardive dyskinesia (5-25.5%) and dystonia (17%) in subjects receiving typical antipsychotic.[118,121–123] Studies have also evaluated the incidence of neuroleptic malignant syndrome[124,134] and extrapyramidal side effects with risperidone.[125] Another study by Pandey *et al*.[136] compared the rate of neuroleptic - induced acute dystonia and reported that there was no difference in vulnerability to develop dystonias in subjects with mania or schizophrenia. With recent interest in weight gain and metabolic syndrome, some of the studies have shown that patients receiving atypical antipsychotics (Olanzapine or risperidone) gain more weight compared to haloperidol and treatment with antipsychotic leads to disturbance in metabolic syndrome parameters[131–133] whereas others suggest contrary findings.[129,130] Studies have also shown that use of typical antipsychotic leads to higher rates of sexual dysfunction[128] and among the atypical antipsychotic sexual desire is more frequently impaired in subjects on risperidone and erectile dysfunction is more prevalent in subjects on olanzapine.[135]

**Table 9:** Side effects of antipsychotics

Author(s)	Methodology	Results
Sarada Menon and Ramachandran[116]	N = 110; Schizophrenia subjects DBRCT, TFD vs. TFP	18/25 receiving TFD developed EPS (Parkinsonism, dystonia, akathesia). Parkinsonism symptoms emerged gradually and mostly after two weeks. Dystonic reaction occurred on the next day after starting the drug in three cases. Akathisia occurred sometimes early and sometimes late. The relationship between clinical improvement and EPS was not found to be significant. 10/20 receiving TFD developed EPS (Parkinsonism, dystonia, akathesia)
Ananth *et al*[117]	N = 960; subjects of psychotic illness receiving phenothiazines in the dose of 75-9010 (mean dose 1228 CPZ equivalent) for 3-14 years	2.9% of the total subjects and 3.4% of the chronic subjects had skin pigmentation
Pandurangi *et al*.[118]	N = 403; Comparative survey Neuroleptics (TFP, FFP, CPZ, HPL)	20 out of 403 (~5%) had been found to be having TD. Prevalence was 3.58% for males and 6.6% for females Age and duration of treatment as a combined variable was a significant predictor of TD. No other variable like duration of treatment, nature of the antipsychotic (TFP, CPZ, FFP), drug combination, dose of drug, whether received antiparkinsonian medications or not, dose of antiparkinsonian medication had significant association with prevalence of TD
Kuruvilla *et al*.[119]	Compared 26 subjects with Schizophrenia (Feingner criteria) with 43 controls; Schizophrenia subjects were receiving either CPZ or TFP for 3 months	Significant elevation in the prolactin level in all schizophrenia subjects after treatment. Female patients had higher rise in prolactin level No significant difference in the prolactin level in the un-medicated subjects with schizophrenia and normal controls
Bhatia *et al*.[120]	60 subjects of schizophrenia received antipsychotics for 3 months	Significant fall in serum calcium and magnesium while other serum electrolytes were unaffected after treatment with drugs or ECT
Ray *et al*[121]	25 subjects, with RDC Schizophrenia or affective dis orders exposed to HPL, TFP, FFP with or without Li	7 out of 25 patients developed akathisia (28%) 3 out of 13 schiz subjects and 4 out of 12 affective disorder subjects developed akathesia. The mean CPZ equivalent dose was not significantly different between the two groups
Dutta *et al*[122]	365 patients on antipsychotics for 3 months	25.5% of subjects on neuroleptics had persistent TD. Increasing age, higher total doses, longer duration of treatment correlated with TD. TD was not correlated to gender
Suresh Kumar and Manoj Kumar[123]	Retrospective comparison of 45 patients with DSM-IIIR schizophrenia vs. 42 patients with mood disorders	The prevalence of extrapyramidal side effects including tardive dyskinesia in the total sample was 71% with equal prevalence in either disorder Extrapyramidal S/Es-dystonia- 17%; akathisia- 25%; TD- 17%
Chopra *et al*.[124]	Retrospective chart review of 4 years data of subjects admitted in intensive care unit with NMS	Incidence of NMS was 1.41 per 1,000 cases treated with neuroleptics and the mortality from NMS was 38% and death occurred at an average of 8^th^ day of developing NMS. All patients who developed NMS received either HPL or TFP Depot FFP was used significantly more often in the group that developed NMS than the control group. Interval between start of therapy and development of NMS ranged from 1 day to 3 years. The group with a fatal outcome had a significantly higher mean age, and mean neuroleptic dose but a lower neuroleptic loading rate Patients who developed NMS received a significantly higher mean neuroleptic dose; more frequently had a H/O concomitant physical or neurological illness, H/O treatment with neuroleptics and developed severe EPS compared to controls
Basu *et al*.[125]	Retrospective review of 43 patients, predominantly schizophrenia on risperidone, mean dose 6.26 mg/d	67.4% had EPS, most patients developed EPS at doses of 5-7 mg/d
Chopra and Raghuram[126]	Retrospective review of 13 cases of NMS, treated with bromocriptine or amantidine	Re-challenge with high potency drugs led to partial recurrence; treatment with single agent better
Gupta *et al*.[127]	Prospective 4-year study	15 patients were found to have NMS of which 3 died; most common diagnosis was mood disorder. HPL used either alone or in combination was the most common culprit. Risk factors -male sex, new exposure, parenteral drugs, rapid dose increases, combination of antipsychotics, concomitant lithium, agitation, dehydration, infections 12 patients recovered completely without any sequelae and APs of different class was reintroduced in 4 of them without recurrence of NMS
Nagaraj *et al*.[128]	Compared sexual dysfunction in 108 subjects of BPAD in remission receiving either typical or atypical antipsychotics	66% of subjects had sexual dysfunction in at least one phase of the sexual response cycle Erectile dysfunction was present in 42% of the sample population and it was the most common type of sexual dysfunction reported. ED was significantly higher with typical than atypical antipsychotics (*P* = 0.025) No significant difference in other aspects of sexual dysfunction across the two groups
Guha *et al*.[129]	Evaluated weight gain and glucose level in 55 subjects receiving OLZ, HPL, TFP for 12 weeks	No significant change in body weight, BMI or plasma fasting glucose in the OLZ group Duration of use of antipsychotic correlated significantly with development of hyperglycemia
Jain *et al*.[130]	80 subjects of Schizophrenia (ICD-10) on OLZ	Patient’s ≥ 40 years of age had significantly higher weight gain. Female Patients ≥ 40 years and all males were prone to gain weight. Females were more likely than males to gain weight. Weight gain was not significantly related to OLZ dose or BMI.
Sahoo *et al*.[131]	Evaluated metabolic parameters of 66 drug naive schizophrenia (DSM- IV) treated with OLZ, Risp or HPL	As per WHO criteria, prevalence of overweight was 22.7% and by IDF criteria prevalence of obesity was 31.8% The prevalence of obesity was 30 times that of matched healthy controls. This was statistically significant Subjects in the Olz group gained 5.1 kg, Risp (4.1 kg) and HPL (2.8 kg) At 6 weeks there was a statistically significant increase in waist circumference, Waist -hip ratio and BMI from baseline to 6 weeks in all groups WC increase, weight gain, BMI: OLZ > Ris*P* > HPL Treatment emergent obesity: 10.3% in OLZ and 9.1% in RISP group none in HPL as per WHO definition and 44.8% in OLZ and 36.4% in RISP group none in HPL as per WHO definition
Sahoo *et al*.[132]	Evaluated metabolic parameters of 30 drug naïve schizophrenia (DSM- IV) treated with OLZ or Risp	Significant increase in systolic and diastolic BP, weight, WC, TG and incidence of metabolic syndrome in the whole sample by 6 weeks Incidence of metabolic syndrome as defined by the IDF criteria, at baseline was 3.33% which increased significantly to 31.81% at 6 weeks No significant change in HDL and FBS levels
Sahoo *et al*.[133]	Evaluated metabolic parameters of 99 drug naive schizophrenia (DSM-IV) treated with OLZ, Risp or HPL	Statistically significant increase in waist circumference, TGL, SBP, HDL levels, FBS levels and DBP from baseline to endpoint between the groups Male subjects have a significant increase in WC, TGL, FBS, SBP and DBP, while female subjects have a significant increase in only WC and DBP Patients with undifferentiated schizophrenia, those with lower BMI scores at baseline gained more weight
Sarkar *et al*.[134]	Prospective study of 672 subjects treated with antipsychotics	3 (0.45%) patients developed NMS. All of them were young males, suffering from mania, and given oral and injectable neuroleptic medications, were agitated, dehydrated and required higher doses of antipsychotics for longer duration.
Nagaraj *et al*.[135]	Compared sexual dysfunction in 102 subjects receiving either Risp, QUET, OLZ	Compared to 23% of healthy subjects with impairment in one or other domain of sexual functioning, 96% in RISP, 88% in QUET and 90% OLZ had some sexual impairment Desire was most commonly impaired in the risperidone group (80%) as compared to 72% in the quetiapine group, and 78% in the olanzapine group. ED was most common in the olanzapine group (50%). Orgasmic dysfunction was equally common to both the risperidone and quetiapine (32%) groups and 27% in the olanzapine group

DBRCT - Double blind randomized controlled trial; TFD - Trifluperidol; TFP - Trifluoperazine; EPS - Extrapyramidal symptoms; TFP - Trifluoperazine; FFP - Fluphenazine; HPL - Haloperidol; TD- Tardive dyskinesia; ECT - Electroconvulsive therapy; RDC - Research diagnostic criteria; S/E - Side effect; NMS - Neuroleptic malignant syndrome; AP - Antipsychotics; BPAD - Bipolar affective disorder; ED - Erectile dysfunction; Olz - Olanzapine; BMI - Body mass index; WHO - World Health Organization; IDF - International diabetes federation criteria for obesity; Risp - Risperidone; BP - Blood pressure; WC - Waist circumference; TG - Triglycerides; HDL - High density lipoprotein; FBS - Fasting blood sugar; SBP - Systolic blood pressure; DBP - diastolic blood pressure; TGL- Triglycerides; Quet- Quetiapine

## CONCLUSION AND FUTURE DIRECTIONS

Reasonably good amount of data on the efficacy of antipsychotics in schizophrenia is available from India. In addition, studies also suggest usefulness of antipsychotics in mania, acute and transient psychosis, delusional disorders and agitation and violence. Older studies also suggest that typical antipsychotics have usefulness in anxiety states. Many of the recent studies followed double blind randomized controlled design and had a reasonable sample size. Further, many studies have been carried out at multiple sites throughout the country. To some extent, data is also available with regards to the tolerability of antipsychotics and it shows that extrapyramidal symptoms and NMS are more common with typical antipsychotics and weight gain is more common with antipsychotics like olanzapine and risperidone.

However, the major limitation of the research is that there are not many studies on the treatment of schizophrenia in elderly, in subjects with comorbid physical illnesses and in cases with comorbid substance abuse. Data on long term efficacy of antipsychotics is also meager. There is still a need to conduct long term comparative multicentric studies to evaluate the efficacy, effectiveness, tolerability and side effects of antipsychotics.
